# Peg-Interferon Lambda Treatment Induces Robust Innate and Adaptive Immunity in Chronic Hepatitis B Patients

**DOI:** 10.3389/fimmu.2017.00621

**Published:** 2017-05-29

**Authors:** Sandra Phillips, Sameer Mistry, Antonio Riva, Helen Cooksley, Tanya Hadzhiolova-Lebeau, Slava Plavova, Krum Katzarov, Marieta Simonova, Stephan Zeuzem, Clive Woffendin, Pei-Jer Chen, Cheng-Yuan Peng, Ting-Tsung Chang, Stefan Lueth, Robert De Knegt, Moon-Seok Choi, Heiner Wedemeyer, Michael Dao, Chang-Wook Kim, Heng-Chen Chu, Megan Wind-Rotolo, Roger Williams, Elizabeth Cooney, Shilpa Chokshi

**Affiliations:** ^1^Institute of Hepatology, Foundation for Liver Research, London, United Kingdom; ^2^Faculty of Life Sciences and Medicine, King’s College London, London, United Kingdom; ^3^Clinic of Gastroenterology and Hepatology, Military Medical Academy, Sofia, Bulgaria; ^4^Johann Wolfgang, Goethe University Medical Center, Frankfurt, Germany; ^5^Oregon Clinical and Translational Research Institute, Portland, OR, United States; ^6^National Taiwan University Hospital, Taipei, Taiwan; ^7^China Medical University Hospital, Taichung, Taiwan; ^8^National Chen Kung University Hospital, Tainan, Taiwan; ^9^University of Hamburg, Hamburg, Germany; ^10^Erasmus University, Rotterdam, Netherlands; ^11^Sungkyunkwan University, Seoul, South Korea; ^12^Hannover Medical School, Hannover, Germany; ^13^Precision Diagnostic Laboratory, Santa Ana, CA, United States; ^14^The Catholic University of Korea, Seoul, South Korea; ^15^Tri-Service General Hospital, Taipei, Taiwan; ^16^Research and Development, Bristol-Myers Squibb, Wallingford, CT, United States

**Keywords:** peg-interferon lambda, direct antiviral, hepatitis B, immunity, *in vivo*

## Abstract

IFN-lambda (IFNλ) is a member of the type III IFN family and is reported to possess anti-pathogen, anti-cancer, and immunomodulatory properties; however, there are limited data regarding its impact on host immune responses *in vivo*. We performed longitudinal and comprehensive immunosurveillance to assess the ability of pegylated (peg)-IFNλ to augment antiviral host immunity as part of a clinical trial assessing the efficacy of peg-IFNλ in chronic hepatitis B (CHB) patients. These patients were pretreated with directly acting antiviral therapy (entecavir) for 12 weeks with subsequent addition of peg-IFNλ for up to 32 weeks. In a subgroup of patients, the addition of peg-IFNλ provoked high serum levels of antiviral cytokine IL-18. We also observed the enhancement of natural killer cell polyfunctionality and the recovery of a pan-genotypic HBV-specific CD4^+^ T cells producing IFN-γ with maintenance of HBV-specific CD8^+^ T cell antiviral and cytotoxic activities. It was only in these patients that we observed strong virological control with reductions in both viral replication and HBV antigen levels. Here, we show for the first time that *in vivo* peg-IFNλ displays significant immunostimulatory properties with improvements in the main effectors mediating anti-HBV immunity. Interestingly, the maintenance in HBV-specific CD8^+^ T cells in the presence of peg-IFNλ is in contrast to previous studies showing that peg-IFNα treatment for CHB results in a detrimental effect on the functionality of this important antiviral T cell compartment.

Clinical Trial Registration: ClinicalTrials.gov NCT01204762.

## Introduction

Type I and type III interferons are the primary mediators of antiviral protection and the main therapeutic protagonists include IFN-alpha (IFN-α) and IFN-lambda (IFNλ; IL-29), respectively. The immune-mediating properties of IFN-α have been extensively described both *in vivo* and *in vitro* in the context of many diseases (1–5). However, little is understood about the immunomodulatory properties of IFNλ in different disease states.

Both type I and III interferons have been shown to play an important role in control of HBV replication (6). Indeed, IFN-α has been used as a treatment strategy for chronic hepatitis B (CHB) for over 40 years; however, its efficacy is suboptimal with resolution of infection being achieved in <7% patients (7, 8). This is marginally improved during combination treatment with potent directly acting antiviral agents, such as entecavir (ETV) or tenofovir, but still remains inadequate with functional cure being achieved in only 15% of patients (9–11). The root cause of this may be immunological in nature. IFN-α has a dual mechanism of action in CHB, first, a direct antiviral effect achieved through inhibiting the synthesis of viral DNA, virus particles, and activation of antiviral enzymes, and second, an augmentation of antiviral host immunity (8). In CHB, IFN-α treatment induces narrowly focused immune responses restricted to activation of the innate immunity with little impact on reactivating stagnant HBV-specific adaptive immune responses which are central to long-term control of infection (12–15).

The precise role and activity of IFNλ as an immunomodulator is unknown *in vivo* in humans and remains unclear in *in vitro* experiments. Indeed, the immune potentiating functions of IFNλ are slowly starting to emerge (16–20). Early data suggests that although IFNλ activates the same signaling pathway as IFN-α, their temporal activation of ISGs as well as the induction of an antiviral response is different (6, 21, 22). There is also some discrepancy regarding the direct impact of IFNλ on immunocytes. Some studies find little or no expression of IFNλR on immune cells, while others show IFNλR expression on both natural killer (NK) and T cells (16, 18, 19, 23, 24). Further to this, IFNλ is also reported to be unable to directly activate NK cell function, influence T cell differentiation, or induce cytokine production by T cells (25–27). In other studies, however, IFNλ stimulates a significant antitumor immunity in murine models (28) and directly modulates T cell activity with promotion of Th1 and inhibition of Th2 responses (16, 29, 30). These discrepancies are likely to be due to differences in the cellular, tissue, and animal models utilized and are compounded by a paucity of studies investigating the relationship between IFNλ and the host immune response *in vivo* (17, 18, 25, 27). Defining whether IFNλ acts as a broad or narrow immunostimulant *in vivo* in the context of a chronic disease will allow its appropriate therapeutic application in infection and disease.

In this study, we have comprehensively analyzed the impact of IFNλ treatment on antiviral immunity in CHB patients. This is an ideal model infection to study the immunostimulatory effects of a therapeutic agent, as persistence of this virus is fundamentally associated with a weak antiviral immune response, characterized by defective NK cells and impaired virus-specific T cell responses (31–38). Moreover, there is strong evidence demonstrating that the development and re-establishment of innate and adaptive host immunity in CHB is associated with control of infection (39–43). Therefore, using CHB infection as a model, we have for the first time utilized the structured platform of a clinical trial to dissect the relationship between the innate and adaptive host immune response and IFNλ.

## Materials and Methods

### Study Design and Patients

**Figure 1:**
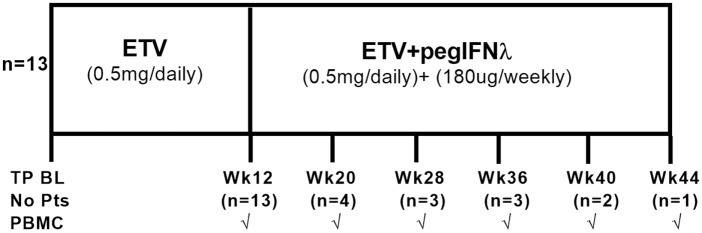
**Study design**. All 13 patients were treated with entecavir (ETV) for 12 weeks and subsequently received ETV + pegIFN-λ. The weeks of treatment reached by the patients are shown. Peripheral blood mononuclear cells (PBMC) collection are also indicated.

We performed longitudinal immuno-surveillance of a subgroup of patients participating in a phase 2b clinical study to evaluate the safety, efficacy and tolerability of pegylated IFNλ (PegIFNλ) in combination with ETV in Hepatitis B e Antigen positive (HBeAg^+^) CHB patients [sponsored by Bristol-Myers Squibb, Wallingford (BMS), CT, USA]. Treatment naïve, HBeAg^+^ CHB patients were recruited in 12 centers world-wide (Portland, California, Frankfurt, Hamburg, Hannover, Rotterdam, Taipei, Tainan, Seoul, and Taichung). The 13 patients (patient characteristics described in Table 1) received 12 weeks of ETV monotherapy (0.5 mg/day) followed by up to 32 weeks of combination therapy ETV (0.5 mg/day)/PegIFNλ (180 μg/weekly) (Figure 1). Clinical parameters [HBV-DNA, HBeAg, hepatitis surface antigen (HBsAg), and alanine aminotransferase (ALT)] were measured in the serum at central laboratories. This study was approved by the Ethics Committee at each recruitment site and informed consent was obtained from all patients before enrollment. The isolation and cryopreservation of peripheral blood mononuclear cells (PBMC) was standardized by supplying each site with a written and video protocol. Prior to patient enrollment, each site performed PBMC isolation dry run which were shipped to the Institute of Hepatology for testing and once PBMCs met standardized criteria of >95% viability and >80% recovery did the sites initiate recruitment and collection of PBMC locally.

**Table 1:** **Patients characteristics at baseline**.

Characteristics	Entecavir and IFN-λ
Age (years)^a^	31.2 (21, 41)
Gender (male/female ratio)	10:3
**Racial group (no. of patients)**	
White	1
Asian*	12
Asian Indian	1
Chinese	4
Korean	4
Other	3
Alanine aminotransferase (IU/ml)^a^	88.2 (38, 297)
HBV-DNA (Log_10_ copies/ml)^a^	8.3 (6.4, 9.7)
**HBV genotype (no. of patients)**	
B	7
C	5
D	1
qHBeAg (Log_10_ copies/ml)^a^	2.4 (0.1, 2.8)
qHBsAg (Log_10_ copies/ml)^a^	4.6 (3.9, 5.4)
**IL-28B (no. of patients)**	
CC	9
CT	4
Non-cirrhotic	13

^a^The data are shown as mean (range). *Asian subgroups are detailed below.

### PBMC Isolation

Peripheral blood mononuclear cells were isolated from heparinized blood by lymphoprep gradient centrifugation as described previously (33, 34, 37). The cryopreserved PBMC were stored at −80°C at each site and subsequently batch shipped to the Institute of Hepatology for immunological analysis.

### Antigens

Commercially available recombinant HBV nucleocapsid protein (HBcAg) and purified HBsAg were purchased from American Research products, Belmont, MA. HBV genotype A, B, C, and D 15-mers overlapping peptides covering the entire HBcAg and HBsAg region (Proimmune, Oxford, UK) were mixed in pools of five adjacent peptides. The pools were reconstituted at 8 mg/ml in dimethyl sulfoxide (DMSO). Recall antigen, tuberculin purified protein derivate (PPD) (Statens Seruminstitut, Copenhagen, Denmark), PMA, inomycin, and phytohemagglutinin (PHA) (Sigma, Poole, UK) were used as positive controls.

### Flow Cytometry

All antibodies used for flow cytometry were purchased from BD Biosciences except when mentioned differently. Cells were acquired after staining on FACSCanto II flow cytometer (BD) and analyzed using FACS DIVA software.

### NK Analysis

Peripheral blood mononuclear cells were surface stained with CD3-BV510, CD16-FITC, CD56-V450, NKG2D-PerCP-eFluor710 (eBioscience), and TRAIL-PE (R&D systems) as previously described (44, 45). To measure the frequency of IFN-γ-producing NK cells and NK cell degranulation, PBMC were incubated for 6 h with rhIL-12 and rhIL-18 (R&D systems) and CFSE-stained K562 (E:T 5:1), respectively. CD107a-APC was added 2 h after the start of the culture to the PBMC: K562 cultures. A protein inhibitor cocktail (eBioscience) was added to all cultures 3 h from the start of the culture. PBMC were then surface stained as described above with CD3, CD16, and CD56 antibodies. The rhIL-12- and rhIL-18-stimulated wells were stained intracellularly with IFN-γ-PE-Cy7 as previously described (44, 45). PBMC were also stimulated with PMA/ionomycin as a positive control. The gating strategy to assess the *ex vivo* frequency of cytokine-producing (CD56^bright^, CD16^−^) and cytotoxic (CD56^dim^, CD16^+^) NK subsets is described in Figure S1 in Supplementary Material.

### Frequency of HBV-Specific Producing T Cells IFN-γ

The frequency was assessed by ELISpot assays. PBMC from patients and from a quality control PBMC batch (inter-assay control) were thawed, washed, and resuspended in RPMI1640/10% AB serum. The cell viability was assessed with propidium iodide using an automated cell counter. ELISPOT assays were performed as previously described (34). PBMC were incubated in the presence of HBcAg (1 µg/ml), HBsAg (2 µg/ml), peptide pools (4 µg/ml), PPD (10 µg/ml), and PHA (2 µg/ml).

### Functions of HBV-Specific T Cells

Peripheral blood mononuclear cells were stimulated with HBV antigens and genotype-specific peptide pools for 7 days. On day 6, PBMC were subjected to a second round of stimulation with the HBV antigens and the overlapping HBV peptides and stained overnight with CD107a-APC and protein inhibitor cocktail. On day 7, PBMC were surface stained with CD3-BV510, CD4-V450, and CD8-APC-eFluor780 antibodies and stained intracellularly for IFN-γ as described above. PBMC stimulated with PMA/ionomycin were used as a positive control.

For T-regulatory cell staining, PBMC were surface stained with CD3, CD4, and CD25-FITC, fixed and permeabilized with FoxP3 buffer (eBioscience) and stained intracellularly with FoxP3-PerCPCy5.5 antibody as per the manufacturer’s instructions.

### Determination of Serum Cytokine Profiles

The serum levels of IL-2, IL-6, IL-8, IL-10, IL-12p70, IL-18, IP-10, IFN-γ, TNF-α, Granzyme B, and MIP-1α were quantitated using cytometric bead array (BD Biosciences) in accordance with manufacturer’s instructions. The levels of IL-15, IL-17, IFN-α, and IFN-β were determined by ELISA (R&D systems).

### Statistics Analysis

Statistical significance was assessed during course of the treatment before and after segregation of patients into groups using repeated measure one-way ANOVA and two-way ANOVA, respectively. Multiple comparisons tests were performed only when the null hypothesis was rejected with the ANOVA test. Pearson’s correlation was used for correlation analyses. Analyses were conducted with the GraphPad Prism software version 6.05 for Windows (La Jolla, CA, USA). *p* < 0.05 was considered statistically significant.

## Results

### Clinical Responses and Group Stratification

This clinical study was terminated early for commercial reasons based on results from a parallel trial showing that non-inferiority of IFNλ to IFNα was not met at week 24. This early curtailment was not related to any safety concerns (46). The 13 patients initially received the full 12 weeks of ETV alone. Due to the truncated nature of this study, four patients received ETV plus Peg-IFNλ for 8 weeks, three for 16 weeks, three for 24 weeks, two for 28 weeks, and one for 32 weeks. Patients’ responses were not significantly different between consecutive time points and were therefore grouped during ETV and ETV plus Peg-IFNλ treatments.

Treatment with ETV alone led to a mean drop of −3.72 Log_10_ copies/ml in HBV-DNA levels during the first 12 weeks of therapy (Table 2) in line with previous studies (47, 48). A further reduction in viral replication was observed with the addition of Peg-IFNλ (−1.8 Log_10_ copies/ml) (Table 2). A significant drop in HBsAg levels (−0.63Log_10_ IU/ml) and in the% HBsAg decline (ETV: 7.8% vs ETV + Peg-IFNλ: 13.2%) was also greater when Peg-IFNλ was administered. HBeAg levels did not fall during ETV alone but the addition of Peg-IFNλ did induce a significant reduction (−0.73Log_10_ IU/ml) and a drop in the % HBeAg decline (ETV + Peg-IFNλ: 31.5%) (Table 2). No HBsAg or HBeAg loss or seroconversion occurred during the course of this truncated study, and no significant changes overall were observed in serum ALT (Table 2). Two patients experienced an ALT flare (ALT greater than 2× baseline and 5× the upper limit of normal) during add-on Peg-IFNλ.

**Table 2:** **Changes in patients virological parameters during treatment**.

Patients (*n* = 13)	HBV-DNA (Log_10_ copies/ml)			Hepatitis surface antigen (HBsAg) (Log_10_ IU/ml)			HBeAg (Log_10_ IU/ml)			Alanine aminotransferase (ALT) (U/l)		
				
	Baseline	Entecavir (ETV)	ETV + pegIFN-λ	Baseline	ETV	ETV + pegIFN-λ	Baseline	ETV	ETV + pegIFN-λ	Baseline	ETV	ETV + pegIFN-λ
M	8.37 ± 0.25	4.65 ± 0.26	2.85 ± 0.27	4.59 ± 0.16	4.23 ± 0.20	3.67 ± 0.35	2.37 ± 0.27	2.32 ± 0.18	1.59 ± 0.32	107.7 ± 29.4	75.82 ± 13.83	114.2 ± 25.84
Δ		−3.72 ± 0.17 (*p* < 0.0001)			−0.29 ± 0.09 (*p* = 0.029)			−0.05 ± 0.16 (*p* = ns)			−31.83 ± 25.47 (*p* = ns)	
Δ1			−5.5 ± 0.23 (*p* < 0.0001)			−0.92 ± 0.23 (*p* = 0.009)			−0.78 ± 0.28 (*p* = ns)			6.53 ± 27.54 (*p* = ns)
Δ2			− 1.8 ± 0.20 (*p* < 0.0001)			−0.63 ± 0.20 (*p* = 0.024)			−0.73 ± 0.23 (*p* = 0.027)			38.37 ± 29.86 (*p* = ns)

*The data are shown as mean ± SEM at baseline (BL), ETV, and ETV + pegIFN-λ. (M) Mean change in HBV-DNA, HBsAg, HBeAg, and ALT from: (Δ) BL to ETV, (Δ1) BL to ETV + pegIFN-λ, (Δ2) ETV to ETV + pegIFN-λ (repeated measures one-way ANOVA was performed)*.

Analysis of the clinical data revealed two distinct groups of patients based on the rates of decline of the viral antigen levels (HBeAg and HBsAg) when Peg-IFNλ was introduced. Nine patients (Group 1) had a greater and significant reduction in HBsAg and HBeAg compared to the remaining four patients (Group 2) who showed no change in viral antigen levels during the addition of Peg-IFNλ (Table 3). In Group 1, HBsAg and HBeAg declined by −0.73 and −0.95 Log_10_ IU/ml, respectively (Table 3). Furthermore, the difference in HBsAg and HBeAg levels between Group 1 and Group 2 was greater than 1 Log (Group 1 − Group 2: HBsAg: −1.07 Log_10_; HBeAg: −1.08 Log_10_) (Table 3).

**Table 3:** **Changes in patients’ virological parameters after segregation in two groups during treatment**.

		HBV-DNA (Log_10_ copies/ml)			Hepatitis surface antigen (HBsAg) (Log_10_ IU/ml)			HBeAg (Log_10_ IU/ml)			Alanine aminotransferase (ALT) (U/l)		
				
		Baseline	Entecavir (ETV)	ETV + pegIFN-λ	Baseline	ETV	ETV + pegIFN-λ	Baseline	ETV	ETV + pegIFN-λ	Baseline	ETV	ETV + pegIFN-λ
Patient Group 1 (*n* = 9)	M	8.14 ± 0.30	4.30 ± 0.30	2.40 ± 0.26	4.39 ± 0.17	4.08 ± 0.19	3.35 ± 0.35	2.21 ± 0.37	2.19 ± 0.24	1.23 ± 0.37	118.5 ± 41.35	70 ± 10.85	112.5 ± 30.87
	Δ		−3.84 ± 0.17 (*p* < 0.0001)			−0.31 ± 0.10 (*p* = ns)			−0.013 ± 0.22 (*p* = ns)			−48.53 ± 35.47 (*p* = ns)	
	Δ1			−5.74 ± 0.24 (*p* < 0.0001)			−1.04 ± 0.32 (*p* = 0.005)			−0.97 ± 0.38 (*p* = 0.019)			−6.07 ± 26.02 (*p* = ns)
	Δ2			− 1.9 ± 0.23 (*p* = 0.0002)			−0.73 ± 0.28 (*p* = 0.042)			−0.95 ± 0.28 (*p* = 0.020)			40.47 ± 27.41 (*p* = ns)

Patient Group 2 (*n* = 4)	M	8.88 ± 0.34	5.43 ± 0.21	3.86 ± 0.21	5.05 ± 0.18	4.81 ± 0.40	4.42 ± 0.28	2.75 ± 0.00	2.65 ± 0.10	2.31 ± 0.32	82.75 ± 26.09	88.25 ± 41.49	117.8 ± 54.54
	Δ		−3.45 ± 0.41 (*p* < 0.0001)			−0.24 ± 0.23 (*p* = ns)			−0.10 ± 0.10 (*p* = ns)			5.5 ± 16.03 (*p* = ns)	
	Δ1			−5.02 ± 0.46 (*p* < 0.0001)			−0.63 ± 0.11 (*p* = ns)			−0.44 ± 0.16 (*p* = ns)			35.03 ± 73.78 (*p* = ns)
	Δ2			−1.57 ± 0.40 (*p* = ns)			−0.39 ± 0.13 (*p* = ns)			−0.33 ± 0.16 (*p* = ns)			29.53 ± 84.19 (*p* = ns)

M Group 1 − M Group 2	Δ3	−0.74 ± 0.47 (*p* = ns)	−1.13 ± 0.47 (*p* = 0.023)	−1.46 ± 0.47 (*p* = 0.041)	−0.65 ± 0.44 (*p* = ns)	−0.73 ± 0.44 (*p* = ns)	−1.07 ± 0.44 (*p* = 0.022)	−0.54 ± 0.46 (*p* = ns)	−0.45 ± 0.46 (*p* = ns)	−1.08 ± 0.46 (*p* = 0.026)	35.77 ± 47.83 (*p* = ns)	−18.26 ± 47.83 (*p* = ns)	−7.21 ± 47.83 (*p* = ns)

*The data are shown as mean ± SEM at BL, ETV, and ETV + pegIFN-λ. (M) Mean change in HBV-DNA, HBsAg, HBeAg, and ALT from (Δ) BL to ETV, (Δ1) BL to ETV + pegIFN-λ, (Δ2) ETV to ETV + pegIFN-λ. (Δ3) Mean difference in HBV-DNA and viral antigens levels between group 1 and group 2 at BL, ETV, and ETV + pegIFN-λ. Repeated measures two-way ANOVA was performed*.

Reductions in viremia were also different between these two groups. Significant reductions were observed in Group 1 between ETV alone and ETV plus Peg-IFNλ (−3.84 Log_10_; −1.9 Log_10_, respectively), whereas HBV-DNA decline was less pronounced in Group 2 (ETV alone: −3.45 Log_10_; ETV + Peg-IFNλ: −1.57 Log_10_). There was a difference in HBV-DNA levels greater than 1 Log between the two groups (ETV alone: −1.13 Log_10_; ETV + Peg-IFNλ: −1.46 Log_10_) (Table 3). Serum ALT levels were not different between Group 1 and 2.

As it has been previously shown that declining HBsAg levels denote activation of the host immunity and control of infection (49–52), the impact of IFNλ on the host immunity was analyzed in the two groups identified: those who did (Group 1) or did not (Group 2) experience changes in antigen levels during combination treatment.

### Addition of Peg-IFNλ Induces a Poly-Functional NK Response

**Figure 2:**
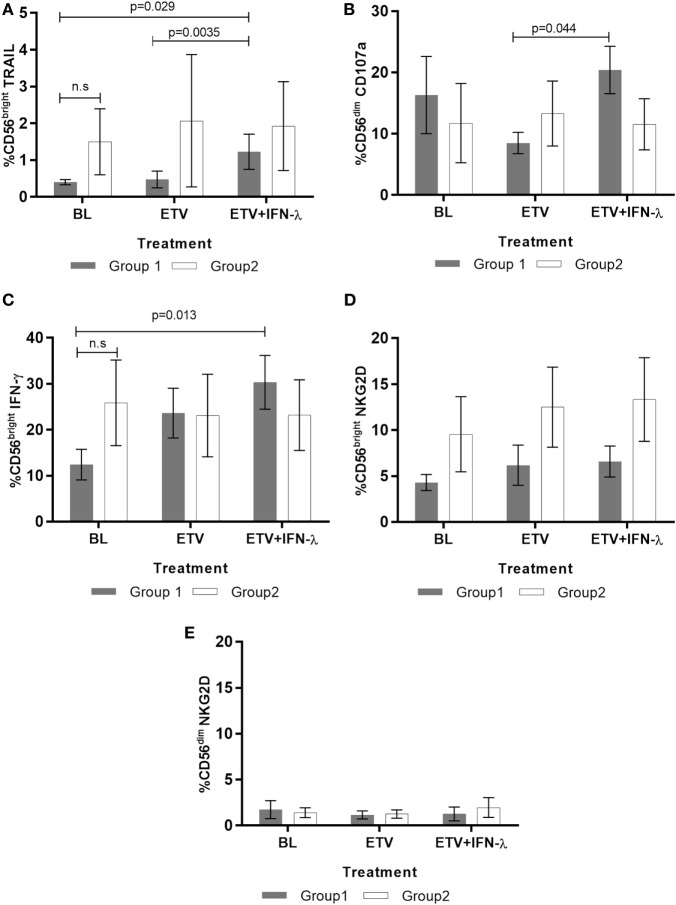
**Effect of treatment on natural killer (NK) cells response in Group 1 and Group 2 patients (*n* = 13)**. Percentage of **(A)** tumor necrosis factor-related apoptosis-inducing ligand (TRAIL)-positive CD56^bright^ NK cells **(B)** CD107a-producing cytotoxic CD56^dim^ NK cells, **(C)** IFN-γ producing CD56^bright^, **(D)** NKG2D-positive CD56^bright^, and **(E)** NKG2D-positive CD56^dim^ were measured by flow cytometry. A total of 100,000 events were collected during FACS acquisition and the subsequent analysis was performed using FACS DIVA software. Data are shown as mean ± SEM. Two-way ANOVA followed by multiple comparison tests were performed for statistical analysis.

During CHB, NK cells exhibit profound impairments in their ability to eliminate HBV by non-cytolytic and cytolytic mechanisms. They notably also lose the ability to orchestrate key players of the adaptive immune response (36, 53–55). Using standardized protocols (44), we analyzed the impact of Peg-IFNλ on the phenotype and functionality of NK cells. In Group 1 patients (i.e., those with the greatest reduction in antigenaemia), we found an expansion in the frequency of these cells expressing the cytotoxic marker tumor necrosis factor-related apoptosis-inducing ligand (TRAIL) during combination Peg-IFNλ (Figure 2A). Importantly, this pattern was not observed in Group 2 patients. We also assessed the relationships between the NK cell population and viral parameters and found the increase in TRAIL-positive NK cells to correlate positively with serum ALT levels in Group 1 patients (*r* = 0.848; *p* < 0.0001). This was reflected in two patients who showed the greatest increase in these NK cells and had the greatest elevation in serum ALT, denoting a cytolytic clearance of infected hepatocytes during the Peg-IFNλ phase of treatment. This observation was in line with previously described findings that in CHB, TRAIL expression increases together with ALT levels in patients treated with Peg-IFNα and denotes the elimination of infected hepatocytes (53). Interestingly, TRAIL-positive cytokine-producing NK cells of Group 1 patients were found to correlate negatively with HBsAg levels (*r* = −0.699; *p* = 0.0025).

The data also clearly demonstrate that NK functionality is modulated by the addition of Peg-IFNλ *in vivo* and is different between the “responding” and “non-responding” groups. The cytotoxic potential of NK cells increased significantly in Group 1 but remained unchanged in Group 2 during the presence of Peg-IFNλ (Figure 2B). The frequency of IFN-γ-producing NK cells also changed during treatment and increased with Peg-IFNλ in Group 1, although the difference between ETV alone and ETV + Peg-IFNλ was not statistically significant (Figure 2C). Nevertheless, the increase in the frequency of IFN-γ-producing NK cells was found to correlate negatively with HBV viral load (*r* = −0.545; *p* = 0.030). In contrast, the expression of the inhibitory marker, NKG2D did not change on any of the NK cells subsets during treatment (Figures 2D,E).

### Peg-IFNλ Augments HBV-Specific T Cells Responses

**Figure 3:**
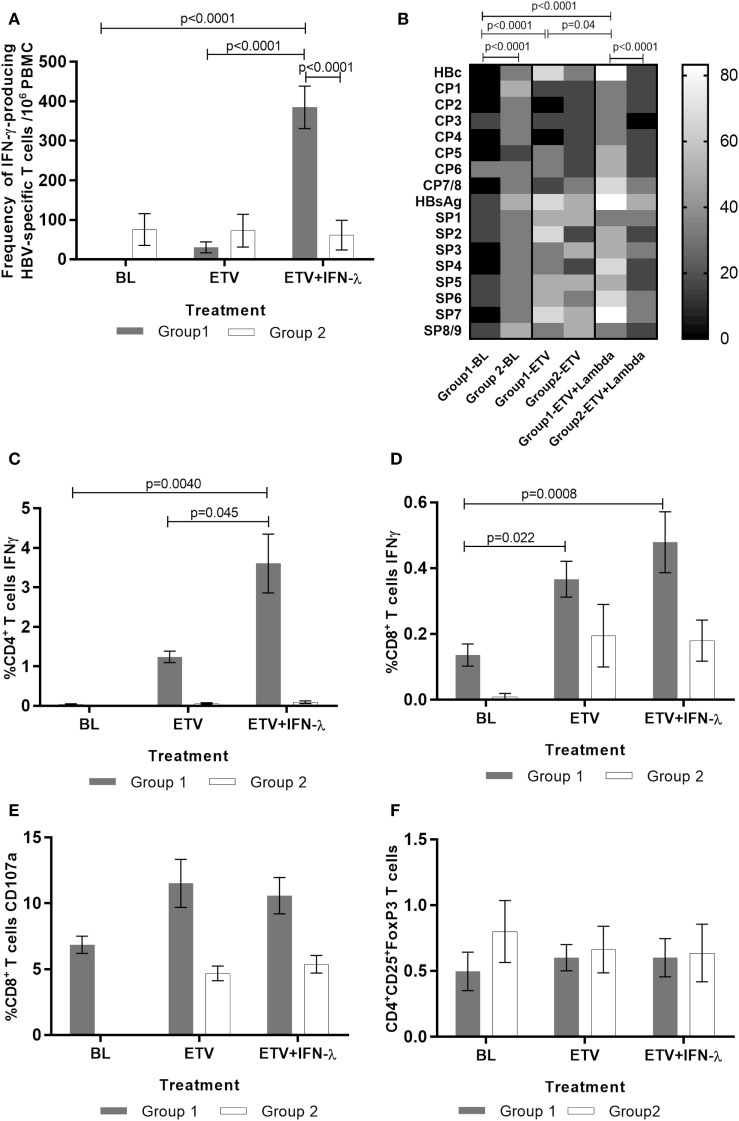
**Effect of treatment on HBV-specific T cells and T regs response in Group 1 and Group 2 patients (*n* = 13)**. The frequency of IFN-γ-producing HBV-specific T cells was evaluated by ELISPOT following peripheral blood mononuclear cells (PBMC) stimulation with HBV antigens and HBV-specific overlapping peptides. PBMC stimulation with recall antigen purified protein derivate and mitogen phytohemagglutinin elicited a measurable strong response which did not change significantly during the course of the treatment. **(A)** ELISPOT quantitation of frequency of IFN-γ-producing HBV-specific T cells. **(B)** Heat map representation of the percentage of patients reacting to each individual HBV antigen and peptide pool in ELISPOT. The assessment of the functionality of T cells was performed by FACS following two rounds of stimulation with HBV antigens and HBV-specific overlapping peptides covering HBV core and HBV surface regions. **(C)** IFN-γ-producing HBV-specific CD4^+^ T cells, **(D)** IFN-γ-producing HBV-specific CD8^+^ T cells, **(E)** CD107a-producing HBV-specific CD8^+^ T cells, and **(F)** T regulatory cells were quantitated by FACS. A total of 100,000 events were collected during FACS acquisition and the subsequent analysis was performed using FACS DIVA software. Data are shown as mean ± SEM. Two-way ANOVA followed multiple comparison tests were performed for statistical analysis.

We comprehensively assessed the HBV-specific T cell response to recombinant HBV core, HBV surface antigen proteins, and overlapping genotype-specific core and surface peptides pools. Significant expansion was observed in the frequency of HBV-specific T cells producing IFN-γ during the add-on Peg-IFNλ phase of treatment in Group 1 but not in Group 2 (Figure 3A). We observed an increase in the percentage of patients who responded to HBV antigens and peptides pools initiated by treatment with ETV alone and this was augmented further when Peg-IFNλ was added (Figure 3B). Further characterization of this reactive T cell population revealed that the increased virus-specific response observed in Group 1 was predominantly driven by the CD4^+^ T cell population (Figure 3C). We also assessed the relationships between CD4^+^ T cell population and viral parameters and found this cell population to be negatively correlated with HBV-DNA (*r* = −0.752; *p* = 0.019) and HBsAg (*r* = −0.795; *p* = 0.010) and positively correlated with ALT (*r* = 0.824; *p* = 0.006). A small but significant increase in the IFN-γ-producing HBV-specific CD8^+^ T cell population was also detected in 40% of patients in Group 1, during ETV and maintained during the addition of Peg-IFNλ (Figure 3D). The change in this cell population was found to correlate negatively with HBsAg (*r* = −0.676; *p* = 0.045) and positively with serum ALT (*r* = 0.770; *p* = 0.015). We also evaluated the cytotoxic potential of HBV-specific CD8^+^ T cells during the study by assessing their ability to degranulate and found that CD107a-positive HBV-specific CD8^+^ T cells were maintained through the treatment period, reflecting the steady levels of ALT observed in most of these patients (Figure 3E). There were, however, higher frequencies of this subset in Group 1 than Group 2. Indeed, during add-on PegIFNλ, 50% of subjects in Group 1 had more than 10% of HBV-specific CD8^+^ T cells expressing CD107a in contrast to none of the patients in Group 2. The frequency of T-regulatory cells was assessed and found to be low in all patients at baseline and did not change during treatment or between the groups (Figure 3F).

### Peg-IFNλ Alters Serum IL-18 Levels

**Figure 4:**
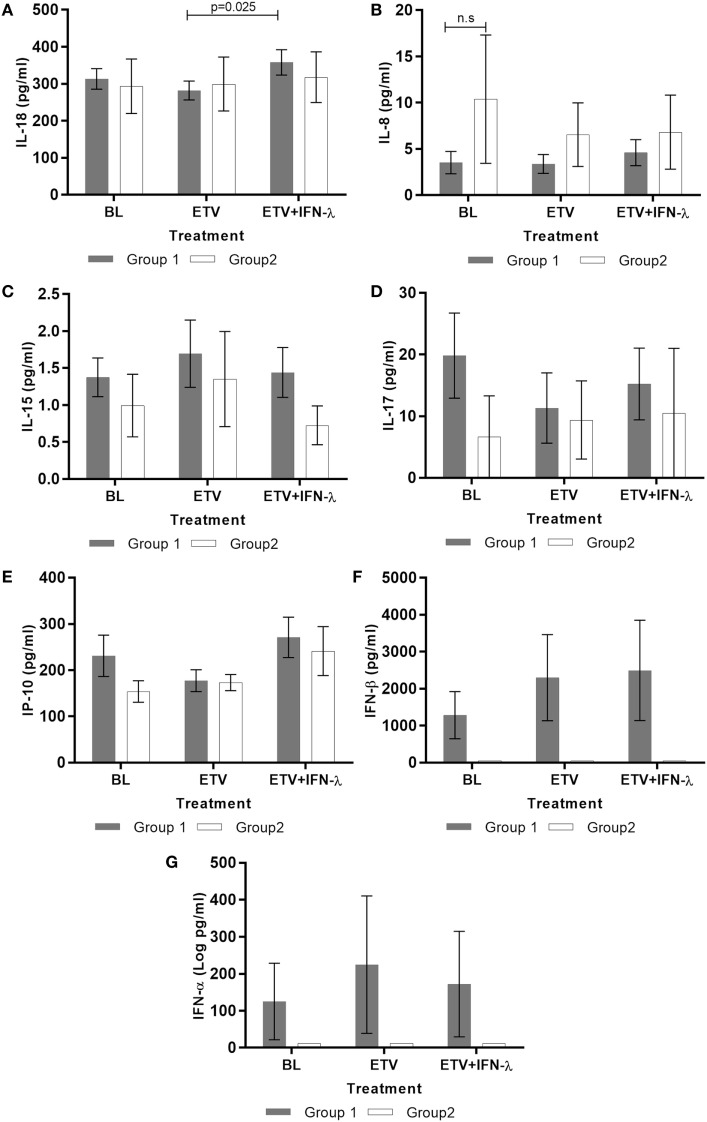
**Effect of treatment on serum cytokines production during treatment in patients group 1 and group 2 (*n* = 13)**. Cytokines **(A)** IL-18, **(B)** IL-8, **(C)** IL15, **(D)** IL-17, **(E)** IP-10, **(F)** IFN-α, and **(G)** IFN-β were measured in the sera of patients by cytometric bead array or ELISA. Data are shown as mean ± SEM. Two-way ANOVA followed by multiple comparison tests were performed for statistical analysis.

Finally, we examined the impact of Peg-IFNλ add-on on a panel of antiviral and pro/anti-inflammatory serum cytokines. Notably, we found that IL-18 levels significantly increased during treatment in Group 1 (Figure 4A). Although this increase is statistically significant, we recognize that it is quite small and the biological relevance needs to be further studied. This change in IL-18 was found to correlate positively with serum ALT (*r* = 0.432; *p* = 0.024). The levels of IL-8, IL-15 IL-17, and IP-10 did not change during the course of treatment in the two groups (Figures 4B–E). Type I IFNs, IFN-β, and IFN-α could be detected but only in Group 1; however, their levels did not change during treatment (Figures 4F,G). The other cytokines measured, IL-2, IL-6, IL-10, IL-12p70, IFN-γ, TNF-α, Granzyme B, and MIP-1α were undetectable in both groups at all time points assessed.

## Discussion

The present data show for the first time that *in vivo*, IFNλ displays immunostimulatory properties and provokes anti-HBV immunity in both the innate and adaptive compartments but only in patients that achieve the greatest decline in viral replication rates. This is of much relevance in CHB, as reductions in, or loss of viremia in the serum does not equate to cure or viral eradication as the HBV genome persists as an integrated genome and/or as episomal covalently closed circular DNA for life (56). Long-term off-treatment control is only likely to be achieved *via* the activation strong antiviral host immunity, as seen in patients who resolve the infection spontaneously (56).

The findings from this study supports our previous reports demonstrating that overcoming immune hyporesponsiveness and development of immune-modulating therapies for CHB can only be achieved in patients who have low viral replication rates (33, 37). This observation is also supported by previous findings from Webster et al. showing that a HBV-DNA load less than 10^7^ copies/ml is the threshold below which circulating multi-specific HBV-specific T cells can be consistently detected (57).

Natural killer cell and virus-specific T cell responses represent the main effectors of a favorable antiviral immune response and are critical in the long-term control of HBV infection (58, 59); functional impairments in their response to HBV have been widely shown to be correlated with an inability of the host to control replication and the persistence of infection (37, 38). In this study, we show that therapeutic administration of IFNλ can induce a functional restoration of NK cells and virus-specific T cells antiviral abilities. Further to this, we show that the spectrum of changes observed with IFNλ treatment are far wider than that observed with conventional IFN-α treatment in CHB patients.

Natural killer cells display two main effector functions that directly contribute to HBV infection control, direct killing of infected cells and the production of a variety of cytokines including the potent anti-HBV cytokine IFN-γ, which has directly antiviral activities and activates and promotes downstream antigen-specific adaptive immune responses (60). In our study, we find an improvement in these two functions of NK cells when Peg-IFNλ is introduced and observe a notable increase of NK cells expressing TRAIL, an activation marker which induces target-cell apoptosis. Studies that investigated the modulation of NK cells during directly acting antiviral therapy have shown no effect on IFN-γ producing CD56^bright^ NK cells; which allow us to conclude that the increase in IFN-γ-producing NK cells observed during add-on Peg-IFNλ is directly the result of IFNλ administration (36, 61). IFNλ mediated activation of cytolytic and non-cytolytic NK functionality is found in our study to be closely associated with reduction in viral replication rates and HBsAg levels. We therefore conclude that IFNλ induces a significant expansion of both cytotoxic and IFN-γ-producing NK cells in patients with the greatest decline in viral replication during ETV.

In addition to the activation of NK cell functionality during IFNλ therapy in Group 1 patients, we also observed significant restoration of the virus-specific T cell responses which are widely known to be pivotal to the host control of HBV replication long-term (43, 62). We observed an increase in the frequency of HBV-specific CD4^+^ and CD8^+^ T cells producing IFN-γ, which correlated strongly with the reduction of viremia and HBsAg. Further to this, we report an increase in the percentage of patients recognizing HBV antigens and peptide pools suggesting a diversification of epitope recognition and T cell activation. This is key for long-term control, as the ability of the immune system to attack multiple targets on a given pathogen has obvious advantages (63). Previous studies of ETV treatment of HBeAg-positive patients have reported partial restoration of HBV-specific CD8^+^ T cells and may explain the small increase in IFN-γ producing HBV-specific CD8^+^ T cells during ETV alone (64, 65). IFN-γ producing HBV-specific CD4^+^ T cells, however, are not susceptible to this ETV-driven immune improvement. In parallel, during IFNλ treatment, we observe a temporal relationship between HBV-specific CD8^+^ T cells and mild elevations of liver transaminases denoting destruction of infected hepatocytes, suggesting the mobilization of activated cytotoxic immune cells into the liver. We have previously shown that this equilibrium between cytolytic and non-cytolytic CD8^+^ T cells functions is critical in control of infection without excessive exacerbation of inflammation and liver injury and this study reveals that IFNλ favorably maintains this balance (66).

It was not possible to delineate the direct mechanisms by which IFNλ activated NK and virus-specific T cell responses. We did explore whether this was mediated *via* the programmed death-1 pathway and found no modulation of the expression of this inhibitory pathway on NK or T cells *ex vivo* during the study period (data not shown). While further work will need to be performed to identify the specific pathways of IFNλ-mediated immune activation, our data does reveal a novel relationship between IFNλ and IL-18, particularly in patients that showed greatest decline in HBsAg levels during IFNλ treatment. This increase in IL-18 levels was however quite small and further studies are needed. The lack of changes in IFN-α and IFN-β plasma levels during IFNλ administration, is in line with previous *in vitro* work by Ank et al (67), suggests that this is also not the mechanism by which host antiviral immunity was induced.

The root cause for lack of immune reactivation in Group 2 could not be fully delineated due to the lack of sample availability. We had hypothesized that hyperexpression of the checkpoint inhibitor programmed death-1 may be partly responsible but this was not substantiated experimentally. This does not preclude the possibility of overexpression of other immune checkpoint inhibitors, on immune cells of group 2 patients, such as Tim-3 and CTLA-4 which have been documented to impair immune function in CHB (68). Further to this, multiple reports have suggested that mutations and splice variants in the HBV genome and lower pregenomic/precore RNA could negatively influence the response to interferon treatment (69–71) and this may also be responsible for lack of response observed in Group 2. Finally and possibly most likely, IFNλ intracellular signaling may have been disrupted by HBV-induced elevated levels of the suppressor of cytokine signaling SOCS 1 and 3 in Group 2 patients, thereby rendering IFNλ treatment ineffective (72, 73). Further in-depth studies addressing these possibilities are required to characterize and confirm the mechanisms underlying IFNλ non-responsiveness *in vivo*.

In this study, we have highlighted several differences in the immunoregulatory activities of IFNλ when compared to IFN-α. The dysregulation of the adaptive immune response, a hallmark of CHB, cannot be overcome by treatment with IFN-α (13). In fact, studies have shown that treatment with IFN-α actively results in the suppression of HBV-specific CD8^+^ T cells (12, 14). It has been hypothesized that this is consequent to the known potent anti-proliferative effects of IFN-α. This suppressive effect of IFN-α is not confined to CHB and has been demonstrated in several other chronic viral infections (74–77). We show that add-on IFNλ treatment does not lead to the suppression but to the maintenance in the frequency of HBV-specific CD8^+^ T cells producing IFN-γ. Their negative strong correlation with HBsAg levels further highlights the importance of these cells in the control of HBV infection. In further contrast, IFN-α does not seem to activate the cytotoxic capacity of NK cells to kill target cells (13), whereas in this investigation we reveal the ability of IFNλ to improve this important effector function. Our data also suggest that IFNλ mediates improvement of anti-HBV immunity *via* IL-18. In contrast, IFN-α is believed to activate NK responses *via* IL-15 (13).

Anti-HBs seroconversion, the marker of functional cure in CHB, was not seen in this study. It is well described that a decline of >1 Log HBsAg is predictive of sustained HBsAg loss in HBeAg-positive CHB patients (78, 79) and we would suggest that given the steady decline of HBsAg levels seen in Group 1 patients during IFNλ treatment, in concert with improvement in innate and adaptive immune responses in Group 1, we may have observed HBsAg loss, possibly followed by anti-HBs seroconversion post-treatment. Additionally, IFNλ was only administered for a truncated 32 weeks and treatment for at least 48 weeks might be needed to observe an on-treatment HBsAg loss and anti-HBs seroconversion especially due to the restricted distribution of IFNλ receptor. Regrettably, there was no posttreatment follow-up due to the early cessation of the clinical trial, due to commercial reasons based on results from a parallel trial showing that non-inferiority of IFNλ to IFN-α was not met at week 24 (46).

In conclusion, this study has demonstrated for the first time a dual immunomodulatory effect of IFNλ on both the innate and adaptive arms of the immune response *in vivo* during chronic viral infection. When IFNλ is administered in patients with suppressed HBV replication rates, it can induce broad immune stimulatory properties and drive activation of cytokine-producing and cytotoxic NK cells, IFN-γ-producing HBV-specific CD4^+^ T and maintenance of the antiviral and cytotoxic functions of HBV-specific CD8^+^ T cells.

## Ethics Statement

The study protocol was approved by the Institutional review board/Independent Ethics Committee at each recruitment site. An informed consent was obtained from all patients before enrolment at each site in accordance with the Declaration of Helsinki.

## Author Contributions

SP performed study design, performed experiments, analyzed results, and manuscript preparation. SM set up assays, conducted the immunological experiments, and acquired data. AR performed statistical analysis. HC performed experiments. SZ, CW, P-JC, C-YP, T-TC, SL, RG, M-SC, HW, MD, C-WK, MS, SP, KK, TH-L, and H-CC recruited patients, collected blood and serum samples, and isolated PBMC. MW-R and EC performed clinical and immunological trial design and manuscript preparation. RW funded the research and edited the manuscript. SC performed study design, analyzed results, and manuscript preparation.

## Conflict of Interest Statement

Megan Wind-Rotolo and Elizabeth Cooney are employed by Bristol-Myers Squibb. The other authors declare no conflict of interest.

## Supplementary Material

Figure S1 **Flow cytometry gating strategy for analysis of CD56^bright^, CD56^dim^ NK cells subsets**. **(A)** The lymphocyte population was gated on forward and side scatter. **(B)** The NK cells population was identified with the CD56 and CD3 markers. **(C)** CD56 and CD16 markers were used to identify CD56^bright^ NK cells (CD56^bright^/CD16^−^) and CD56^dim^ NK cells (CD56^dim^/CD16^+^) subpopulations. A total of 250,000 events were acquired. Analysis was performed using FACS diva software.
