# Supplementary figures and images for: Peg-Interferon Lambda Treatment Induces Robust Innate and Adaptive Immunity in Chronic Hepatitis B Patients
Source: Front Immunol. 2017 May 29;8:621. doi: 10.3389/fimmu.2017.00621 (PMC5446997; doi:10.3389/fimmu.2017.00621)

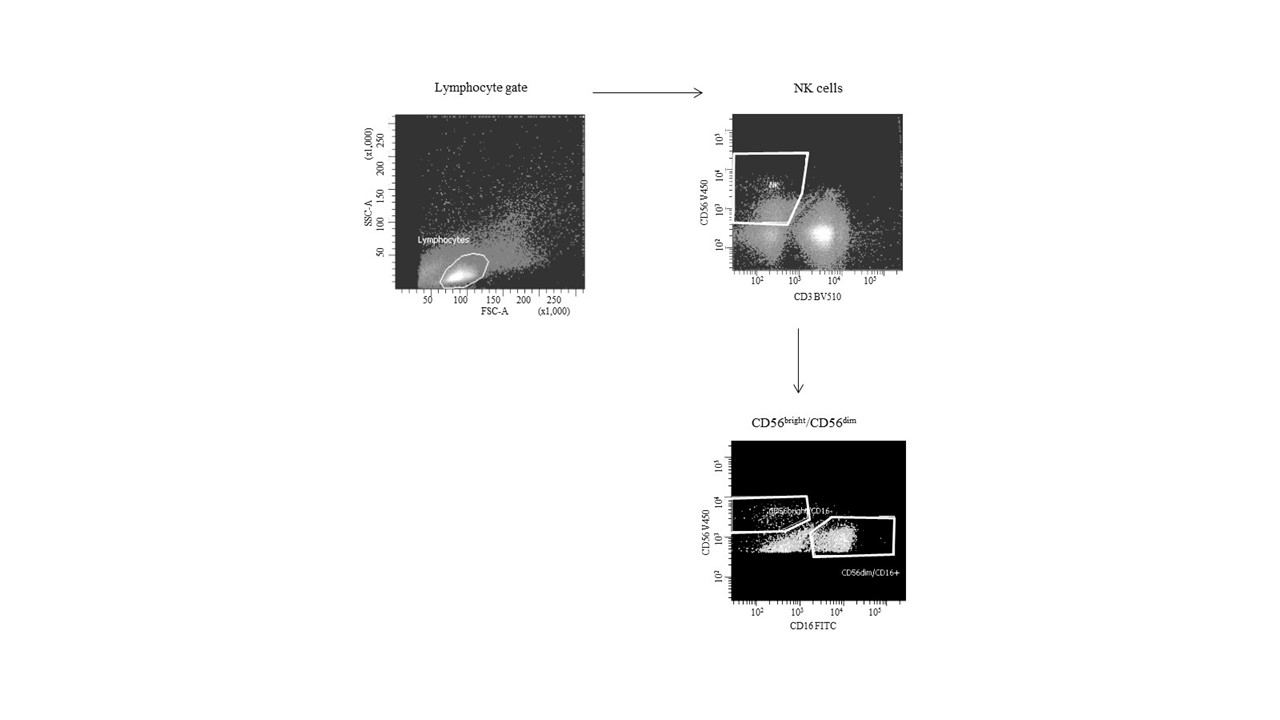

Supplement: Figure S1 — Flow cytometry gating strategy for analysis of CD56bright, CD56dim NK cells subsets. (A) The lymphocyte population was gated on forward and side scatter. (B) The NK cells population was identified with the CD56 and CD3 markers. (C) CD56 and CD16 markers were used to identify CD56bright NK cells (CD56bright/CD16−) and CD56dim NK cells (CD56dim/CD16+) subpopulations. A total of 250,000 events were acquired. Analysis was performed using FACS diva software. [file Image_1.JPEG]
